# Calcium Intake and Serum Concentration in Relation to Risk of Cardiovascular Death in NHANES III

**DOI:** 10.1371/journal.pone.0061037

**Published:** 2013-04-10

**Authors:** Mieke Van Hemelrijck, Karl Michaelsson, Jakob Linseisen, Sabine Rohrmann

**Affiliations:** 1 Cancer Epidemiology Group, Division of Cancer Studies, School of Medicine, King's College London, London, United Kingdom; 2 Department of Surgical Sciences, Uppsala University, Uppsala, Sweden; 3 Institute of Epidemiology I, Helmholtz Zentrum München, Neuherberg, Germany; 4 Institute of Social and Preventive Medicine, University of Zurich, Zurich, Switzerland; Universidad Peruana de Ciencias Aplicadas (UPC), Peru

## Abstract

**Background:**

Evidence for an association between calcium intake and risk of cardiovascular death remains controversial. By assessing dietary intake, use of supplements, and serum levels of calcium, we aimed to disentangle this link in the third National Health and Nutrition Examination Survey (NHANES III).

**Methods:**

Mortality linkage of NHANES III to death certificate data for those aged 17 years or older (n = 20,024) was used to estimate risk of overall cardiovascular death as well as death from ischemic heart disease (IHD), acute myocardial infarction (AMI), heart failure (HF), and cerebrovascular disease (CD) with multivariate Cox proportional hazards regression analysis.

**Results:**

About 10.0% of the population died of cardiovascular disease and the majority (5.4%) died of IHD. There was increased risk of overall CVD death for those in the bottom 5% of serum calcium compared to those in the mid 90% (HR: 1.51 (95% CI: 1.03–2.22)). For women there was a statistically significant increased risk of IHD death for those with serum calcium levels in the top 5% compared to those in the mid 90% (HR: 1.72 (95%CI: 1.13–2.61)), whereas in men, low serum calcium was related to increased IHD mortality (HR: 2.32 (95% CI 1.14–3.01), P_interaction_: 0.306). No clear association with CVD death was observed for dietary or supplemental calcium intake.

**Conclusions:**

Calcium as assessed by serum concentrations is involved in cardiovascular health, though differential effects by sex may exist. No clear evidence was found for an association between dietary or supplementary intake of calcium and cardiovascular death.

## Introduction

A large number of studies have investigated the potential link between serum calcium levels and cardiovascular death [1], [2], [3], [4], [5], [6], [7], [8]. Already in 1996, Reunanen and colleagues [7] found an increased risk of premature death in men (<50 years) with increasing serum calcium levels. They examined mortality rate in relation to a single serum calcium measurement obtained at screening of 33,346 persons during a mean follow-up period of 10.8 years [7]. In contrast, a meta-analysis focused on chronic kidney disease patients found no association between all-cause mortality and serum levels of calcium [6], but a German prospective study of 1206 patients with coronary heart disease found a strong positive association between serum calcium and all-cause mortality [1].

Calcium supply is usually assessed through evaluation of calcium intake via diet or supplements. A recent study by Li and colleagues, using data from the Heidelberg cohort of the European Prospective Investigation into Cancer and Nutrition (EPIC), showed no association between dietary intake of calcium or use of calcium supplements and cardiovascular mortality [9]. These findings were in conjunction with what was found in another large prospective study [10], but a Swedish population-based prospective study of men with relatively high intakes of dietary calcium showed a reduction in all-cause mortality when taking more than the recommended daily amount [11]. With respect to calcium supplementation, Bolland and colleagues reanalyzed the Women's Health Initiative Calcium/Vitamin D Supplementation Study (WHI CaD Study) based on 36,282 postmenopausal women and found no statistically significant association of calcium supplements with cardiovascular death [12]. In a related meta-analysis, the authors combined clinical trials comparing calcium supplements versus placebo and found no association with overall death [12]. Nevertheless, the majority of studies focused on women and few studies investigated different types of cardiovascular death or a dose-response relationship [9], [12], [13], [14], [15], [16]. The most recently published study from the National Institutes of Health –AARP Diet and Health Study showed that during a mean follow-up time of 12 years, there was an excess risk of CVD death related to high intake of supplemental calcium in men, but not in women [8].

Thus, the current evidence for an association between calcium supply, serum calcium levels and risk of cardiovascular death remains controversial. Calcium is not only required for contraction and relaxation of heart muscles, but is also a second messenger in signal transduction pathways of the cardiovascular system. An imbalance of normal calcium homeostasis has been linked to both heart failure and hypertension, but a complex array of mechanisms still needs to be disentangled [17]. By assessing dietary intake, use of supplements, as well as serum levels of calcium, we aimed to study this link in the third National Health and Nutrition Examination Survey (NHANES III) by evaluating overall cardiovascular death as well as death due to ischemic heart disease (IHD), acute myocardial infarction (AMI), heart failure (HF), and cerebrovascular disease (CD) in both men and women.

## Methods

### Study population and data collection

The NHANES III Mortality linkage provides follow-up data from the date of NHANES III survey participation (1988–1994) through December 31, 2006. The latter is a cross-sectional study conducted by the National Center for Health Statistics (NCHS) between 1988 and 1994 [18] and was designed as a multistage stratified, clustered probability sample of the US civilian non-institutionalized population at least two months old. The included subjects participated in an interview conducted at home and an extensive physical examination, which included a blood sample, taken in a mobile examination center and participants were randomly assigned to participate in either the morning or afternoon/evening examination session [18]. In total, 30,818 people were interviewed in NHANES III and had a physical examination and a blood sample taken. The NCHS has updated the mortality linkage of NHANES III to death certificate data obtained in the National Death Index (NDI) such that mortality ascertainment is based on results of a probabilistic match between NHANES III and NDI death certificate records [19]. From this linkage, we selected all men and women eligible for mortality follow-up (all participants 17 years or older), who did not have a history of heart disease (n = 18,714).

Ionized serum calcium was quantified with NOVA 7 analyzer (Nova Biomedical, Waltham, MA), a Hitachi 737 Analyzer (Boehringer Mannheim Diagnostics, Indianapolis, IN) [20], [21]. Since calcium is tightly regulated we categorized ionized serum calcium based on the cut-offs for the bottom 5% and top 5%, i.e. <1.16, 1.16–1.31, 1.31+ mmol/L which is consistent with what is indicated in the literature as normal ionized serum calcium [22]. Dietary calcium was evaluated in all individuals with one 24-h dietary recall. A validation study involving a repeated 24-h recall for 5% of the respondents showed that the correlation between the two measurements was 0.46 for men and 0.40 for women, suggesting a moderate day-to-day variation in calcium intake [23], [24]. Using the recommended dietary allowance of 1000–1300 mg per day as a reference [25], we categorized daily dietary calcium intake as follows: <500, 500–1000, 1000–1300, >1300 mg. Calcium supplements were calculated based on the information obtained in the vitamin/mineral supplementation questionnaires, which provide information on the dietary supplement code and daily dosage. By merging this information with the Supplement Concentrations documentation provided by NHANES III, we estimated the mean daily dosage of calcium per individual. In addition to a dichotomous covariate indicating whether a person took any calcium supplementation or not, we categorized calcium supplement intake using 2000 mg as the maximum tolerable dosage [25] (i.e. <500, 500–1000, 1000–2000, 2000+mg) and also quantified how often a supplement was taken per month. Due to sample size issues, the first two groups of calcium supplement intake were combined for the stratified analyses. Finally, we calculated to the total daily calcium intake by adding the dietary and supplement intake of calcium per day. The information was categorized as <500, 500–1000, 1000–1300, 1300–2000, and >2000 mg with 1000–1300 mg/day as the reference.

Other covariates in the analysis included age, race/ethnicity, poverty-to-income-ratio, comorbidity index, smoking, alcohol consumption, physical activity, serum vitamin D, total energy intake (kcal), body mass index (BMI), waist circumference, and serum C-reactive protein (CRP). Race and ethnicity were combined into four race–ethnic groups: Non-Hispanic white, Non-Hispanic black, Mexican American and Other. The poverty to income ratio is an index of poverty status which is calculated by dividing family income by a poverty threshold specific to family size. Comorbidity was evaluated with a comorbidity coefficient similar to the Charlson Comorbidity Index (CCI), as used in other NHANES III-based analyses [26]. Each of the comorbidities (i.e., history of heart attack, congestive heart failure, diabetes, hypertension, etc.) available in the dataset contributed one point to the composite index with additional points given for older age. Cigarette smoking, alcohol consumption, physical activity, and total energy intake were assessed using questionnaires. 25-hydroxy vitamin D was measured using the Diasorin radioimmunoassay kit (Diasorin, Stillwater, MN, USA) on frozen serum from 1994 to 1995. Coefficients of variations from quality control samples ranged from 13% to 19%. The radioimmunoassay kit was calibrated using high-performance liquid chromatographically purified vitamin D every six months. Body mass index (BMI) was calculated as weight in kilograms divided by the square of height in meters. The first was measured to the nearest 0.01 kg with an electronic digital scale while the participant was wearing foam slippers and paper shirt and trousers, whereas height was measured to the nearest 0.1 cm using a stadiometer. Waist circumference was measured at the iliac crest to the nearest 0.1 cm. Finally, serum CRP, an established inflammatory biomarker, was measured using a low sensitivity method that can detect levels >3.0 mg/L [27]. The protocols for the conduct of NHANES III were approved by the institutional review board of the NCHS, Centers for Disease Control and Prevention. Written informed consent was obtained from all participants [27].

### Data analysis

The association between dietary calcium intake and serum ionized calcium as well as the association between calcium supplement intake and serum ionized calcium was evaluated using a linear regression model adjusted for age and sex. Sampling weights for NHANES III were used to account for sampling variability and to adjust for differential probability of selection of persons [18]. To evaluate the association between different measurements of calcium (serum, dietary intake, and supplements) in relation to cardiovascular death, we used multivariate Cox proportional hazards regression. Cardiovascular death (CVD) was defined by ICD-10: I00–I99, and was subdivided into IHD (ICD-10: I20–25), AMI (ICD-10: I21–22), HF (ICD-10: 50), and CD (ICD-10: I60–69). All models were adjusted for age, sex, race/ethnicity, poverty to income ratio, comorbidity index, smoking, alcohol consumption, physical activity, BMI, and serum vitamin D. The models for dietary and supplement calcium intake were also adjusted for total energy intake. Stratified analyses were conducted for men and women, as well as for levels of vitamin D (</≥20 ng/mL [28]). We tested for interaction of calcium measurements with sex or vitamin D concentration using an interaction term for the respective categorical variables. The latter stratification was performed since vitamin D deficiency has been linked to cardiovascular disease and has a key role in calcium homeostasis [29], [30]. Due to small number of events these stratified analyses were only performed for overall CVD and IHD death. To graphically display Hazard Ratios (HRs) representing the dose-response association for daily intake of calcium supplements and cardiovascular death, we used the Restrictive Cubic Spline (RCS) function of daily calcium supplement and dietary intake with 4 knots (0.05, 0.35, 0.65, and 0.95 percentile [31]) in a multivariate Cox proportional hazards model as described above. This analysis was performed using the RCS_Reg SAS Macro created by Desquilbet and Mariotti [32]. All tests were two-sided; p-values <0.05 were considered to be statistically significant. All analyses were conducted with Statistical Analysis Systems (SAS) release 9.2 (SAS Institute, Cary, NC).

## Results

About 10.0% of the population died of cardiovascular disease and the majority (4.9%) died of IHD. All baseline characteristics of the study population are shown in Table 1.

**Table 1:** Baseline characteristics of study population by vital status.

		Death due to:				
	No death	Any CVD	IHD	Acute MI	Heart Failure	Cerebr Disease
		(*ICD10:I00*–*I99*)	(*ICD10: I20*–*I25*)	(*ICD10:I21*–*22*)	(*ICD10: I50*)	(*ICD10: I60*–*69*)
*N*(*%*)	*16844* (*90.01*)	*1870* (*9.99*)	*1006* (*5.38*)	*362* (*1.93*)	*133* (*0.71*)	*352* (*1.88*)
**Mean age** (SE), years	40.65 (0.35)	68.85 (0.73)	68.68 (0.78)	65.51 (1.16)	74.02 (1.43)	69.43 (1.91)
**Sex**						
Men	47.46	45.72	50.69	56.00	41.00	36.50
Women	52.54	54.28	49.31	44.00	59.00	63.50
**Race-Ethnicity**						
Non-Hispanic white	75.10	82.49	83.61	85.16	87.26	81.09
Non-Hispanic black	11.24	11.32	9.95	8.64	9.89	12.39
Mexican American	5.49	2.55	2.52	2.39	1.06	3.17
Other	8.18	3.64	3.92	3.80	1.79	3.34
**Poverty to income ratio**						
<1.4	19.18	27.23	26.89	35.95	30.33	22.71
1.4–3.17	34.42	35.73	35.88	36.17	38.14	36.99
≥3.17	46.40	37.04	37.23	37.88	31.53	40.31
**Mean follow-up time** (SE), months	173.29 (2.67)	98.74 (2.71)	97.14 (2.37)	98.57 (4.22)	115.42 (5.32)	95.22 (6.44)
**Ionized serum calcium**, mmol/L						
Mean (SE)	1.24 (0.003)	1.23 (0.004)	1.23 (0.004)	1.23 (0.005)	1.23 (0.007)	1.23 (0.008)
Bottom 5% (<1.16)	3.17	6.04	7.54	5.91	3.36	3.26
Mid 90% (1.16–1.31)	91.56	86.70	84.58	86.87	93.88	87.94
Top 5% (1.31+)	5.27	7.26	7.89	7.27	2.76	8.80
**Dietary calcium intake** (SE), mg						
<500	30.33	37.77	38.97	38.08	39.51	33.45
500–1000	38.55	40.62	41.01	42.67	40.62	44.13
1000–1300	13.47	11.83	11.94	10.43	8.01	12.67
>1300	17.65	9.78	8.06	8.82	11.85	9.76
**Calcium supplement users**	19.31	18.14	18.09	16.36	20.54	18.91
**Daily supplemental calcium intake**, mg						
0	79.37	81.10	81.21	82.75	79.46	80.36
0–500	17.41	14.77	15.04	12.40	13.11	15.51
500–1000	2.24	2.74	2.53	3.93	4.23	3.61
1000–2000	0.81	1.20	1.21	0.92	3.20	0.52
≥2000	0.17	0.20	0.00	0.00	0.00	0.00
**How often supplement taken per month** (SE)	6.86 (0.34)	6.72 (0.54)	6.54 (0.76)	6.31 (1.18)	8.36 (2.74)	6.53 (1.28)
**Total calcium intake**						
<500	30.21	42.86	42.44	40.29	51.26	39.87
500–1000	36.10	35.40	37.03	38.32	30.22	36.86
1000–1300	13.53	10.76	11.30	10.39	6.28	11.78
1300–2000	14.57	8.09	6.02	8.53	8.07	10.90
>2000	5.60	2.87	3.21	2.48	4.17	0.59
**History of Hypertension**	34.23	76.21	75.68	71.03	78.36	77.33
**History of Diabetes**	3.95	14.14	16.10	19.79	9.42	12.78
**Mean Serum Vitamin D** (SD), ng/mL	29.87 (0.36)	26.43 (0.42)	26.52 (0.61)	27.30 (0.95)	26.33 (1.42)	26.86 (1.00)
**Comorbidity Index**						
0	0.53	0.03	0.01	0.00	0.00	0.07
1	67.03	34.06	33.91	33.78	39.58	36.27
2	24.16	40.12	40.82	40.54	38.53	34.60
3+	8.27	25.78	25.26	25.68	21.89	29.06
**Alcohol consumption**						
Never	43.64	63.81	63.19	66.80	64.76	64.61
Up to once a week	20.00	14.59	13.57	15.92	24.58	14.11
2–3 times a week	14.49	4.25	4.77	4.69	2.65	3.59
4–6 times a week	12.06	5.27	5.32	3.62	2.96	4.39
Daily or more	9.81	12.09	13.16	8.96	5.06	13.29
**Smoking behaviour**						
Never	48.20	43.89	43.37	40.11	54.43	47.86
Former	23.14	32.72	30.81	32.80	28.18	34.14
Current	28.66	23.39	25.83	27.08	17.39	18.00
**Vigorous Physical activity**	9.54	26.28	26.89	26.81	32.86	26.83
**Total energy intake** (SE), kcal	2257.18 (18.67)	1729.61 (28.85)	1721.82 (35.87)	1697.40 (66.03)	1686.41 (86.78)	1661.49 (70.67)
**BMI** (kg/m^2^)						
<18.50	2.79	3.25	2.83	1.12	5.03	3.19
18.50–24.99	45.02	36.58	37.16	34.64	35.38	39.87
25.00–25.99	31.41	33.39	32.64	36.93	31.52	34.26
>30.00	20.78	26.78	27.37	27.31	28.07	22.68
**Mean Waist circumference** (SE), cm	90.46 (0.26)	97.78 (0.66)	98.28 (0.86)	98.50 (1.17)	96.95 (2.06)	95.25 (1.37)
**Mean CRP** (SE), mg/L	3.85 (0.09)	6.06 (0.30)	6.10 (0.49)	4.68 (0.44)	6.51 (0.84)	6.30 (0.82)

The majority of deaths occurred among non-Hispanic whites (82.5%). The poverty-to-income ratio was rather equally distributed among those who died, with slightly smaller proportion of people in the lowest tertile of the ratio (27.2%). The proportion of people with serum calcium in the bottom or top 5% was higher for those who died of cardiovascular disease compared to those who did not (Table 1).

When put together in the same age- and sex-adjusted model, neither dietary nor supplement intake were associated with serum calcium levels (results not shown), nor did we find an association with more extreme (bottom and top 5%) values of dietary, total or supplement intake (results not shown).

Table 2 shows the hazard ratios (HRs) for cardiovascular death using different measurements of calcium. The fully adjusted model shows an increased risk of overall CVD death for those in the bottom 5% of serum calcium compared to those in the mid 90% (HR: 1.51 (95% CI: 1.03–2.22)). The association was also observed for death due to IHD and AMI, though with lower precision. High serum calcium (top 5%) was also, although not statistically significantly, associated with increased IHD mortality (HR: 1.32 (95% CI 0.96–1.81)).

**Table 2:** Hazard Ratios and 95%Confidence Intervals for risk of cardiovascular death by serum, dietary, supplement, and total calcium.

	Hazard Ratios and 95%Confidence Intervals (CI) for Death due to:				
	Any CVD	IHD	Acute MI	Heart Failure	Cerebr Disease
	(*ICD10:I00*–*I99*)	(*ICD10: I20*–*I25*)	(*ICD10:I21*–*22*)	(*ICD10: I50*)	(*ICD10: I60*–*69*)
**Ionized serum calcium** 1, mmol/L					
<1.16	1.51 (1.03–2.22)	1.94(1.27–2.94)	1.67 (0.86–3.20)	0.64 (0.15–2.67)	0.81 (0.38–1.71)
1.16–1.31	1.00 (Ref)	1.00 (Ref)	1.00 (Ref)	1.00 (Ref)	1.00 (Ref)
1.31+	1.16 (0.85–1.58)	1.32 (0.96–1.81)	1.23 (0.71–2.11)	0.40 (0.14–2.20)	1.35 (0.67–2.72)
**Dietary calcium intake** 2 (SE), mg					
<500	1.03 (0.75–1.41)	1.13 (0.74–1.71)	1.32 (0.76–2.30)	1.61 (0.59–4.37)	0.79 (0.37–1.68)
500–1000	0.98 (0.76–1.26)	1.01 (0.69–1.46)	1.20 (0.80–1.79)	1.40 (0.52–3.73)	0.96 (0.53–1.74)
1000–1300	1.00 (Ref)	1.00 (Ref)	1.00 (Ref)	1.00 (Ref)	1.00 (Ref)
>1300	0.90 (0.59–1.35)	0.71 (0.38–1.31)	0.80 (0.41–1.55)	1.75 (0.45–6.83)	0.87 (0.37–2.07)
**Calcium supplement users** 2 (versus no supplements)	0.84 (0.67–1.04)	0.88 (0.68–1.14)	0.85 (0.56–1.31)	0.81 (0.32–2.01)	0.79 (0.45–1.36)
**Daily supplemental calcium intake** 2, mg					
0	1.00 (Ref)	1.00 (Ref)	1.00 (Ref)	1.00 (Ref)	1.00 (Ref)
0–500	0.85 (0.66–1.09)	0.90 (0.68–1.19)	0.80 (0.46–1.37)	0.71 (0.25–2.06)	0.81 (0.50–1.32)
500–1000	0.71 (0.49–1.13)	0.73 (0.40–1.32)	1.23 (0.53–2.82)	0.51 (0.07–4.01)	0.81 (0.20–3.20)
1000–2000	0.92 (0.59–1.44)	0.95 (0.42–2.15)	1.15 (0.37–3.54)	2.64 (0.57–12.13)	0.42 (0.08–2.16)
≥2000	1.62 (0.27–9.75)	NA	NA	NA	NA
**Total daily calcium intake**, mg					
<500	1.07 (0.77–1.49)	1.07 (0.71–1.61)	1.30 (0.76–2.22)	1.89 (0.70–5.13)	0.86 (0.47–1.56)
500–1000	1.07 (0.82–1.38)	1.08 (0.75–1.56)	1.34 (0.87–1.76)	1.54 (0.59–4.05)	1.00 (0.59–1.69)
1000–1300	1.00 (Ref)	1.00 (Ref)	1.00 (Ref)	1.00 (Ref)	1.00 (Ref)
1300–2000	0.86 (0.59–1.25)	0.60 (0.35–1.03)	0.91 (0.42–1.95)	1.51 (0.40–5.68)	1.04 (0.49–2.25)
>2000	1.01 (0.52–1.95)	1.07 (0.44–2.58)	0.82 (0.34–1.96)	2.43 (0.50–11.91)	0.19 (0.05–0.76)

1 Adjusted for: age, sex, race/ethnicity, poverty to income ratio, comorbidity index, serum vitamin D, alcohol consumption, smoking behaviour, vigorous physical activity, and BMI.

2 Adjusted for: age, sex, race/ethnicity, poverty to income ratio, comorbidity index, serum vitamin D, alcohol consumption, smoking behaviour, vigorous physical activity, total energy intake, and BMI.

**Figure 1:**
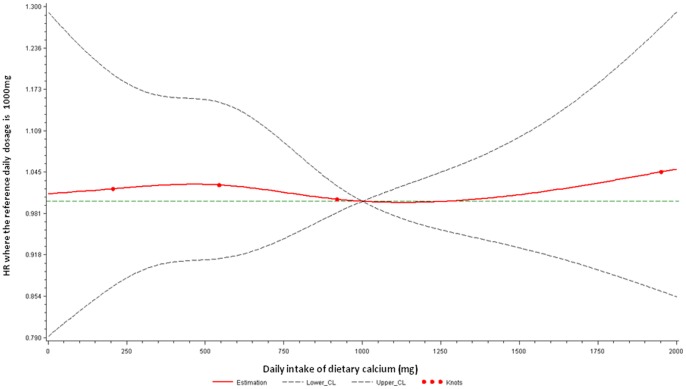
Adjusted dose-response association between daily dietary calcium intake and risk for cardiovascular death. Calcium intake was coded using an RCS function with four knots arbitrarily located at the 0.05, 0.35, 0.65, and 0.95 percentile. Y-axis represents the adjusted hazard ratio for cardiovascular death for any value of calcium supplement/dietary intake compared to individuals with 1000 mg per day intake. Dashed lines are 95% confidence intervals. Knots are represented by dots.

Dietary calcium intake was not associated with an increased risk of cardiovascular death (Table 3). Changing the categories of dietary calcium (<178, 178–348, 348–539, 539–764, 764–1105, 1105–1789, 1789+ mg/day) into more extreme groups did not alter these findings (results not shown). The dose-response association between dietary calcium intake and cardiovascular death is also illustrated in Figure 1.

**Table 3:** Hazard Ratios and 95%Confidence Intervals for risk of cardiovascular death by serum, dietary, supplement, and total calcium, stratified by sex.

	Hazard Ratios and 95%Confidence Intervals (CI) for Death due to:				P for interaction	
	MEN		WOMEN			
	Any CVD	IHD	Any CVD	IHD	Any CVD	IHD
	(*ICD10:I00*–*I99*)	(*ICD10: I20*–*I25*)	(*ICD10:I00*–*I99*)	(*ICD10: I20*–*I25*)	(*ICD10:I00*–*I99*)	(*ICD10: I20*–*I25*)
**Ionized serum calcium** 1, mmol/L						
<1.16	1.85 (1.14–3.01)	2.32 (1.39–3.88)	1.23 (0.72–2.08)	1.53 (0.69–3.39)		
1.16–1.31	1.00 (Ref)	1.00 (Ref)	1.00 (Ref)	1.00 (Ref)	0.637	0.306
1.31+	0.93 (0.55–1.57)	0.87 (0.46–1.67)	1.33 (0.88–1.99)	1.72 (1.13–2.61)		
**Dietary calcium intake** 2 (SE), mg						
<500	1.23 (0.79–1.90)	1.13 (0.66–1.96)	0.86 (0.58–1.28)	1.13 (0.57–2.21)		
500–1000	1.06 (0.81–1.40)	0.99 (0.66–1.47)	0.86 (0.59–1.26)	1.03 (0.53–1.99)		
1000–1300	1.00 (Ref)	1.00 (Ref)	1.00 (Ref)	1.00 (Ref)	0.025	0.525
>1300	0.78 (0.50–1.22)	0.60 (0.28–1.31)	1.10 (0.61–1.97)	0.96 (0.43–2.15)		
**Calcium supplement users** 2 (versus no supplements)	0.81 (0.54–1.22)	0.78 (0.47–1.29)	0.85 (0.64–1.12)	0.98 (0.70–1.36)	0.580	0.403
**Daily supplemental calcium intake** 2, mg						
0	1.00 (Ref)	1.00 (Ref)	1.00 (Ref)	1.00 (Ref)	0.555	0.439
0–1000	0.78 (0.50–1.21)	0.75 (0.43–1.32)	0.90 (0.69–1.19)	1.07 (0.79–1.45)		
1000–2000	0.89 (0.32–2.45)	1.01 (0.32–3.22)	0.65 (0.33–1.30)	0.67 (0.29–1.53)		
≥2000	1.44 (0.37–5.66)	0.91 (0.13–6.67)	0.82 (0.54–1.25)	0.93 (0.38–2.28)		
**Total daily calcium intake** 2, mg						
<500	1.25 (0.79–1.97)	1.05 (0.61–1.82)	1.23 (0.86–1.74)	1.41 (0.83–2.39)	0.102	0.589
500–1000	1.14 (0.83–1.57)	1.03 (0.68–1.56)	1.01 (0.69–1.47)	1.20 (0.67–2.14)		
1000–1300	1.00 (Ref)	1.00 (Ref)	1.00 (Ref)	1.00 (Ref)		
1300–2000	0.81 (0.52–1.26)	0.49 (0.25–0.98)	0.85 (0.49–1.47)	0.73 (0.35–1.52)		
>2000	1.01 (0.44–2.29)	0.98 (0.34–2.85)	0.84 (0.44–1.62)	0.92 (0.32–2.62)		

1 Adjusted for: age, race/ethnicity, poverty to income ratio, comorbidity index, serum vitamin D, alcohol consumption, smoking behaviour, vigorous physical activity, and BMI.

2 Adjusted for: age, race/ethnicity, poverty to income ratio, comorbidity index, serum vitamin D, alcohol consumption, smoking behaviour, vigorous physical activity, total energy intake, and BMI.

**Figure 2:**
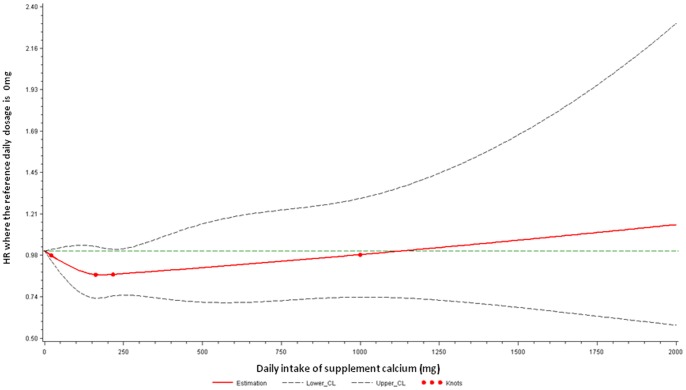
Adjusted dose-response association between daily calcium supplement intake and risk for cardiovascular death. Calcium supplement intake was coded using an RCS function with four knots arbitrarily located at the 0.05, 0.35, 0.65, and 0.95 percentile. Y-axis represents the adjusted hazard ratio for cardiovascular death for any value of calcium supplement/dietary intake compared to individuals with 0 mg per day intake. Dashed lines are 95% confidence intervals. Knots are represented by dots.

Calcium supplement use was unrelated to the risk of CVD death (Table 2). Additional adjustment for frequency of calcium supplement usage during the past month or waist circumference instead of BMI did not alter these estimates (results not shown). Stratification by vitamin D levels did not alter any of the findings observed in Table 2 nor did excluding CCI from the models (results not shown). The dose-response association between supplemental calcium intake and cardiovascular death is also illustrated in Figure 2.

In a sex-stratified analysis (Table 3), slightly different patterns were observed for men and women. For women there was a statistically significant increased risk of IHD death for those with serum calcium levels in the top 5% compared to those in the mid 90%, whereas in men, serum calcium in the bottom 5% was related to increased CVD and IHD mortality. Moreover, there was also a statistically significant decreased risk of IHD death for men with a total daily intake 1300–2000 mg/day compared to those with total daily intake 1000–1300 mg/day, but there was no association for calcium intake from diet only. However, the only statistically significant interaction was seen for dietary calcium intake and sex with an increased risk of CVD mortality for men with a low intake (<1000 mg/day) compared to those with intake 1000–1300 mg/day in contrast to a protective effect for women with low dietary calcium intake (P_interaction_ = 0.025).

## Discussion

This large cohort study evaluated the association between calcium intake, serum calcium levels and different types of cardiovascular death in both men and women, while taking into account a large number of potential confounders, including serum vitamin D levels. Overall, we found an increased risk of cardiovascular death when serum calcium levels were <1.16 mmol/L compared to 1.16–1.31 mmol/L, but for women, there was an increased IHD mortality among women with high serum calcium levels (>1.31 mmol/L). No associations were found between cardiovascular death and dietary or supplement calcium intake. However, there was a protective effect for total calcium intake of 1300–2000 mg/day and death from IHD among men. No obvious differences were observed by serum levels of vitamin D.

Calcium is known to be part of the aetiological pathways of cardiovascular disease. For instance, coronary artery calcium scores specify the presence of calcium in coronary arteries and are indicative of atherosclerotic plaques and thus risk of cardiovascular disease [33]. Nevertheless, calcium has mainly been studied in relation to bone health and only more recently cardiovascular safety was raised as a concern in osteoporosis management [34]. Two recent meta-analyses showed an increased risk of up to 31% for incident AMI when comparing calcium supplement users versus placebo [13], [35]. In addition to calcium supplements, also dietary intake and serum levels of calcium have been studied in relation to incident cardiovascular disease. The most recent study, based on the Heidelberg cohort of EPIC, showed an inverse association between dietary and dairy calcium intake and risk of AMI, but an increased risk among users of calcium supplements [9].

All the above studies focused on incident cardiovascular disease and suggest a role for calcium, nevertheless the findings for an association with cardiovascular mortality have been less consistent with very few studies investigating both serum levels as well as dietary and supplement intake in both men and women [9], [34]. At an annual meeting of the American Association of Clinical Endocrinologists it was recently concluded, based on a review of all relevant articles published between 1992 and 2011, that there is inconsistent evidence for an association between calcium supplementation ≥500 mg/day and an increase in cardiovascular mortality risk [36]. Moreover, in a cohort of patients with stable coronary heart disease it was shown that high serum calcium levels were strongly associated with mortality risk (HR for 4^th^ versus 1^st^ quartile: 2.39 (95% CI: 1.22–4.66)) [1]. However, the association between dietary calcium and cardiovascular death was evaluated in a cohort of 23,366 Swedish men aged 45–79 years who did not take any dietary supplements and showed a non-statistically significant lower rate of cardiovascular death (HR: 0.77 (95% CI: 0.58–1.01) when comparing the highest intake tertile (≥1,599 mg/day) to the lowest (<1,230 mg/day) [11]).

Our study thus assessed three different measurements of calcium and partly corroborates previous findings. In terms of serum calcium, we found a positive association between low serum calcium levels and cardiovascular death, which was confined to men, as well as high levels and IHD death for women. This finding may have been obscured in previous studies due to larger serum calcium categories or the focus on a specific patient population such as chronic kidney disease patients [6]. Since the test for interaction by sex was not statistically significant this finding may simply be due to chance. We did not find an overall association between dietary or supplement calcium intake and cardiovascular mortality, which is in contrast with the most recent study based on the NIH AARP-Diet and Health study [8]. This study and our study both adjusted for a wide range of potential confounders and have comparably long follow-up, but it is possible that differences in assessment of supplement intake explain the inconsistent findings between both studies.

The main strength of this study is its generalizibility as it uses nationally representative data including both men and women. In addition, we were able to take into account many potential confounding factors and perform stratified analyses by sex and vitamin D levels. Repeated measurement could have strengthened the accuracy of different calcium measurements as a single measurement may be prone to measurement error and within-person variation. Dietary calcium intake was assessed using a single 24-hour recall, which may not reflect a participant's habitual diet. Supplement intake in NHANES III is self-reported but the recording of the supplement name from the label is a strength compared with many other studies. Nevertheless, given the complexity of supplements data collection may be subject to some error and the analytic verification of the supplement's actual content would be required to accurately measure the levels of calcium intake [37]. Despite its relative large sample size, we lacked power for some of the stratified analyses and could not perform a stratified analysis for the association between dietary or supplement calcium intake and cardiovascular mortality by serum calcium levels according to the extreme cut-offs used.

## Conclusion

The current study suggests that calcium is involved in cardiovascular health. However there was no strong evidence for an association between calcium supplement usage and cardiovascular death indicating that calcium supplement usage may be a marker of healthy life style despite adjustments for comorbidities, smoking, alcohol consumption, and levels of physical activity. However, it is possible that calcium supplementation may have a different effect on incident cardiovascular disease than on fatal cardiovascular disease. Whether extremely low or extremely high serum concentrations of calcium are related to cardiovascular mortality, in particular IHD, and whether these associations differ by sex needs to be addressed in future studies in which these markers of calcium are assessed in detail.
